# The expanding impact of T-regs in the skin

**DOI:** 10.3389/fimmu.2022.983700

**Published:** 2022-09-15

**Authors:** Edries Yousaf Hajam, Patricia Panikulam, Chung-Ching Chu, Haarshadri Jayaprakash, Amitabha Majumdar, Colin Jamora

**Affiliations:** ^1^ IFOM ETS- The AIRC Institute of Molecular Oncology Joint Research Laboratory, Centre for Inflammation and Tissue Homeostasis, Institute for Stem Cell Science and Regenerative Medicine (inStem), Bangalore, Karnataka, India; ^2^ School of Chemical and Biotechnology, Shanmugha Arts, Science, Technology and Research Academy (SASTRA) University, Thanjavur, Tamil Nadu, India; ^3^ Unilever Research & Development Shanghai, Shanghai, China; ^4^ Unilever Research & Development Bangalore, Bangalore, India

**Keywords:** skin, skin immunology, skin disease, regulatory T (Treg) cells, inflammation, innate immunity, adaptive immunity

## Abstract

As the interface between the body and the environment, the skin functions as the physical barrier against external pathogens and toxic agents. In addition, the skin is an immunologically active organ with a plethora of resident adaptive and innate immune cells, as well as effector molecules that provide another layer of protection in the form of an immune barrier. A major subpopulation of these immune cells are the Foxp3 expressing CD4 T cells or regulatory T cells (T-regs). The canonical function of T-regs is to keep other immune cells in check during homeostasis or to dissipate a robust inflammatory response following pathogen clearance or wound healing. Interestingly, recent data has uncovered unconventional roles that vary between different tissues and we will highlight the emerging non-lymphoid functions of cutaneous T-regs. In light of the novel functions of other immune cells that are routinely being discovered in the skin, their regulation by T-regs implies that T-regs have executive control over a broad swath of biological activities in both homeostasis and disease. The blossoming list of non-inflammatory functions, whether direct or indirect, suggests that the role of T-regs in a regenerative organ such as the skin will be a field ripe for discovery for decades to come.

## Skin as an immune organ

The skin is an immunologically active organ that harbors multiple immune effector molecules and cells which belong to both innate and adaptive branches of immunity. Apart from its role as a physical barrier protecting the body from the external environment, the skin through the activity of immune cells and molecules, forms the first line of natural immunological defence against infection (1).

The skin is a multi-layered structure, and each layer contains cells and effector molecules that contributes towards the mounting of an immune response and hence infection protection. The epidermis for example contains Langerhans cells and T cells, which, can be found in the stratum basale and stratum spinosum. Apart from this, the dermis harbours dendritic cell (DC) subsets, including dermal DCs and plasmacytoid DCs (pDCs), and T cell subsets, including CD4+ T helper 1 (TH1), TH2 and TH17 cells, γδ T cells natural killer T (NKT) cells and Tregs.

In addition to the immune cells linked to an adaptive immune response, innate immunity activators are also present in the skin. Key innate immune activators present in the skin layers are the TLRs, NLRs, cytokines, chemokines, antimicrobial peptides, and lipids. In addition, macrophages, mast cells and fibroblasts and keratinocytes are also present in the skin tissue. All these acts in a concerted fashion and they co-ordinate a strong immune response against invading pathogens. Skin keratinocytes also produce antimicrobial peptides and lipids as a part of its natural innate immune response against pathogens. The production of things like antimicrobial peptides (AMPs) and lipids (AMLs) forms a part of a natural and evolutionarily conserved defence mechanism of eukaryotic cells against bacteria and viruses. AMPs and AMLs prevent microbial infection of skin by direct killing of the pathogen, recruitment of host immune cells and modulation of cytokine production (2).

The optimal regulation of this skin resident immune system and its effectors molecules (AMPs, AMLs) is thus important for skin health and hygiene and maintenance of such an activity is driven by the efficient crosstalk between the skin cells, immune cells and effectors and the microbes. In the following sections of this review, we will cover in the key role played by the immune cells in maintaining this with a focus on the role of the Tregs.

## Cyclical regulation of cutaneous immune cells under homeostatic conditions

Most of the T regulatory cells isolated from human skin expresses CCR4. It is also shown that a good percentage of peripheral blood CD4+ CD25^hi^ FOXP3+ T-regs express cutaneous lymphocyte antigen (CLA). These cells were shown to be capable of immunosuppressing activated CD3+ T cells suggesting that they might be recruited to maintain immune tolerance in human skin in homeostatic conditions (3). FACS analysis of healthy human skin have shown that a major portion of T-regs in human skin are memory T-regs as they express CD45RO. These cells were at one point activated due to the expression of CTLA-4. There are reduced number of T-regs in fetal skin compared to adult skin. Immunofluorescence analysis have shown that in human skin, T-regs reside close to follicular epithelium compared to interfollicular dermis whereas conventional T cells are distributed throughout the dermis. More T-regs are present in the areas where there is high density of hair like the scalp. Memory T-regs are non-migratory as they lacked CCR7 (4).

**Figure 1:**
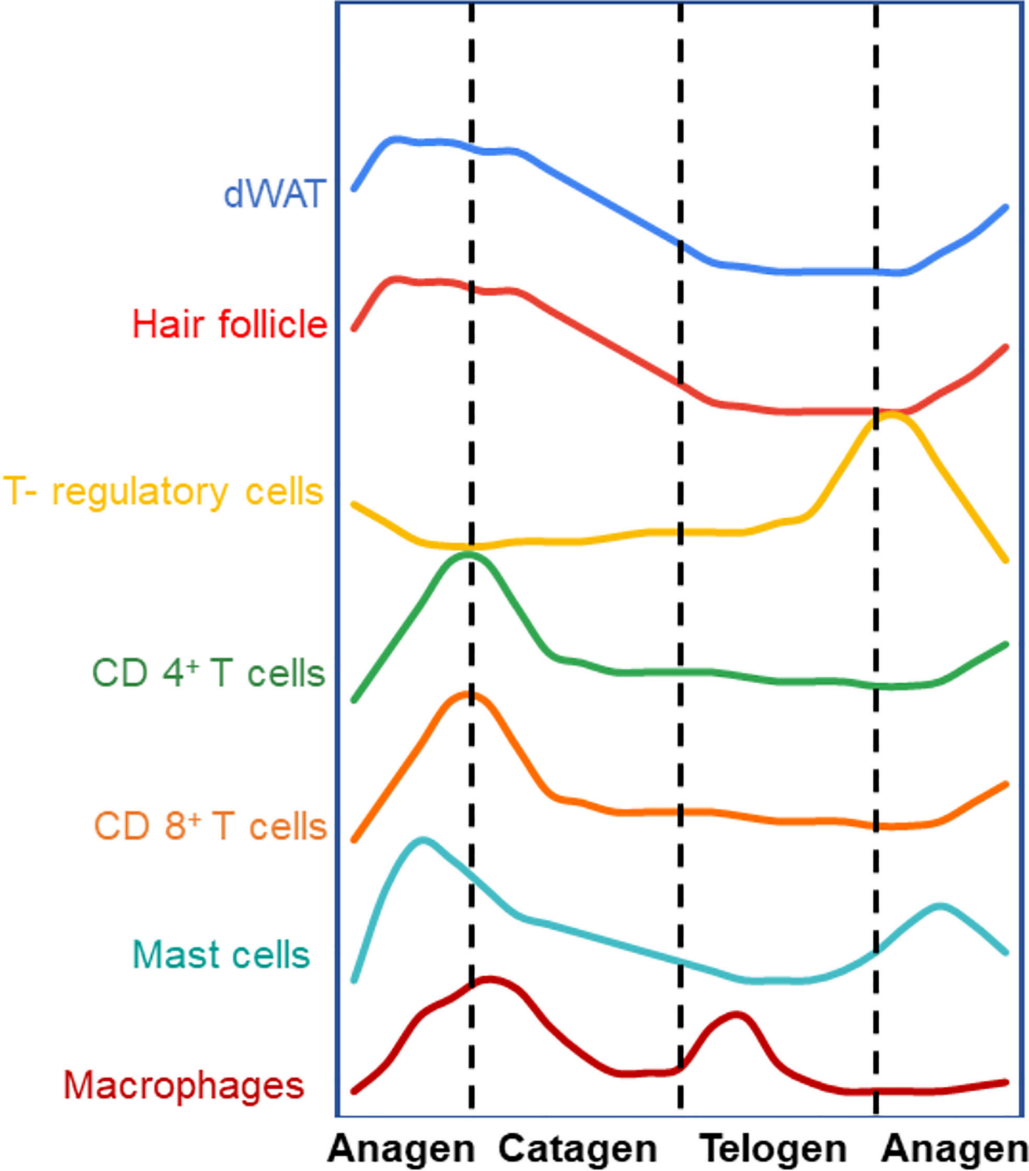
Different compartments of the skin cycle under homeostatic conditions (Arbitrary levels). The hair follicle is well known to undergo regenerative cycles of anagen (growth), catagen (regression) and telogen (resting phase). Immune cells and dermal white adipose tissue (dWAT) cycle synchronously with respect to different stages of hair follicle. Most of the immune cells follow a similar trend where they increase in number during anagen and decline with catagen and telogen. Interestingly, T-regs cycle in the opposite fashion – T-regs are more in number in the telogen phase than anagen or catagen. This could signify a possible regulatory relationship between these different cell types though mechanisms governing this cyclical behavior are yet to be defined. (5–16).

Under homeostatic conditions, the hair follicles undergo cycles of growth (anagen), regression (catagen) and quiescence (telogen) throughout life (**Figure 1**). In rodents, hair growth commences in the embryo and continues postnatally followed by catagen and telogen phases. The early growth phase is called morphogenesis and it starts from the anterior and progresses to the tail in rodents. Interestingly, unlike rodent hair follicles that cycle synchronously for the first two cycles (5, 6), the hair follicles in human skin cycle independently from the adjacent hair follicle. The stem cells in various niches of the hair follicle proliferate and differentiate to maintain the hair follicle unit, and requires communication between the epithelial compartment and the mesenchymal compartment (17). The hair follicles and the dermal white adipose tissue (dWAT) expand during anagen and shrink during the catagen and telogen stages.

dWAT is layer of white adipose tissue which resides in the dermal layer skin under the fibroblasts and above the muscle layer panniculus carnosus (18). Well know function of dWAT is the thermoregulation but recent studies have unearthed importance of dWAT in antimicrobial peptide secretion (19), wound healing, and hair follicle cycling (7, 20). Similar to the dynamics of the hair follicle and dWAT, many resident immune cells cycle in both numbers and activity in the skin (**Figure 1**). This concurrent cycling between immune cells with the different cutaneous structures suggests that there may be a regulatory and functional link between them. For instance, macrophages cycle in sync with the hair follicle. By IF and FACS analysis of murine back skin, it was shown that macrophages gradually increase in number after the commencement of the anagen phase and attains a peak at mid telogen, followed by a significant decrease in the late telogen phase due to apoptosis and migration (8). Interestingly, these changes in macrophage number along with their release of Wnt7b and Wnt10a during apoptosis are necessary for induction of the anagen phase of the hair follicle (8). During the late anagen phase, an increased number of a subset of macrophage-like cells expressing FGF5 was observed (9, 10). These cells then migrate to the lower dermal region where they interact with the FGFR1 on dermal papillae to induce the regression of hair follicle marking the beginning of catagen phase.

Similar to macrophages, mast cells numbers and degranulation of its stored cytokines also in sync with the hair follicle cycle (**Figure 1**). The mast cell population accumulates towards the end of anagen and just before catagen and decrease in numbers during telogen. Around 70% of mast cells at very early anagen are degranulated whereas only 5.8% are degranulated in telogen skin (11, 12). It has been shown that mast cell degranulation is essential for HF cycling, primarily in at the anagen phase (12) through its secretion of factors such as adrenocorticotropic hormone, and Substance P (13).

In addition to the innate immune cells, some adaptive immune cells also cycle under homeostatic conditions in the skin. CD4+ and CD8+ T cells, expressing the αβ TCR, spontaneously appear in the early neonatal skin (6). In the epidermis of murine dorsal skin, the highest number of CD4^+^ T-cells are observed at postnatal day 3 followed by a gradual decline in number. CD8^+^ T cells are the prime T cell population in the epidermis, whereas the CD4^+^ T cell population dominates the dermis (6). Analysis of murine skin by immunofluorescence staining over different stages of the hair cycle has shown that CD4+ T cells and CD8+ T cells are higher in number in the anagen skin when compared to telogen skin. In both depilation and cyclosporin induced hair cycling, the higher number of these T-cells in anagen vs telogen is exaggerated. As with innate immune cells, this trend is opposite with respect to T-regs in skin (6, 14–16). Interestingly, despite the cyclic fluctuations in the number of these cells, the ratio of CD4+/CD8+ T-cells remains constant in steady state (6).

Recently new roles of T-regs in regulating the homeostasis of various skin compartments that undergo cyclical rounds of regeneration have been reported. For instance, T-regs themselves undergo cyclic changes in numbers as well as in activation markers (such as; CD25, ICOS, Ki67, CTLA-4, and GITR) with respect to the cyclic changes in hair follicle, dWAT and other immune cells (**Figure 1**) (16). Consistent with their immune suppressive activities, cutaneous T-regs are generally higher when innate and adaptive immune cells numbers and activity are low and vice versa. It is also interesting to note that only the T-regs in close proximity to the hair follicle stem cell region showed an active (amoeboid) morphology. On the other hand, T-regs further away from the hair follicle showed a more spherical morphology indicative of a lack of activity. This could indicate spatial differences in T-reg activity and function with respect to other cells within the skin (16). We expect that the increasing use of single cell analysis will facilitate the further understanding of novel roles of T-regs during cyclic regeneration of the skin.

## Introduction to cutaneous T-regs

Given its role as a master regulator of other immune cells, T-regs may play an executive role in the cellular dynamics of other immune cells thereby contributing to the cyclic changes in the skin directly or indirectly. The discovery of T-regs was reported in the mid-90’s as a subpopulation within the pool of CD4+CD25+ T-cells. CD25 was established as a marker of T-regs (21), with a lot of skepticism, since activated T effector (T-eff) cells highly express CD25. However, the skepticism started to dissipate with the discovery of CD45RB expression on T-regs, which helped to distinguish the T-regs from the T-eff cells (22). Finally, the discovery of a new specific marker forkhead box transcription factor FOXP3 in 2003 settled the debate over the unambiguous existence of T-regs (CD4+ CD25+ FOXP3+ T-regs). The expression of FOXP3 is both necessary and sufficient for T-reg immune suppressive activity (23–26) and its absence leads to development of severe lymphoproliferative autoimmune disease in mice (Scurfy mouse) and in humans (IPEX syndrome) (27, 28). On a cautionary note, recent studies show that the FOXP3 can be expressed in various mammalian cell types during embryonic stages and only becomes restricted to T-regs in adults. Thus, it is difficult to perform lineage tracing experiments in embryogenesis using FOXP3 alone to understand T-reg development in the skin (29).

These T-regs, referred to as natural T-regs (nT-regs), arise from the thymus where they acquire the ability to distinguish self from non-self-antigens (30, 31). Studies from thymectomized mice, which develop lymphoproliferative disease, demonstrated that the transfer of healthy CD4+ cells (which includes both T-eff and T-regs) prevented the development of this phenotype. This study also revealed that IL2 is important in the maturation of T-regs by stabilizing FOXP3 expression. Later it was found that CD4 T cells can be induced to become T-regs (induced T-regs or iT-regs) in the peripheral tissues as well as *in vitro* cultures by secreted factors such as retinoic acid and TGF beta (32–34).

T-regs are a heterogenous group of cells both in lymphoid and non-lymphoid tissues. Single cell analysis of mouse T-regs from these two groups of tissues suggested that skin T-regs resembled colonic T-regs (35), though interpretations should be predicated on the low number of cells captured. Nevertheless, the sequencing data revealed that the chemokine receptors *Ccr4*, *Ccr8*, and *Cxcr4* were upregulated in both colon and skin T-regs, while *Ccr6* was specific to the skin T-regs. They also found shared expression of genes in the skin and colonic T-regs such as *Gata3*, *Il1rl1*, *Tnfrsf4*, *Rora* (35). More than 95% of T-regs in adult skin express CD45RO, which indicates that the cells have previously encountered an antigen outside the thymus (36). Skin T-regs have higher levels of the T-reg activation markers such as CTLA4, CD25, ICOS and Foxp3 compared to T-regs in peripheral blood. A switch in expression (either up or down) of 812 genes was discovered along the brachial lymph node to skin migration. Initially, T cell migration and glycolytic process help in the adaptation of T-regs in skin followed by cell proliferation; cytokine production and fatty acid homeostasis. The adaptation of T-regs in mouse skin was conserved in human skin T-regs as well.

There is evidence that suggest that T-regs express the transcription factors of the target cells they are attempting to suppress. For example, M.A. Koch et al., showed in 2009 that in response to interferon-γ, mouse T-regs upregulated the T helper 1 (Th1)-specifying transcription factor T-bet, which is required for the homeostasis and function of T-reg cells during Th1-mediated inflammation (37). Similarly, it was observed that interferon regulatory factor-4 (IRF4), a transcription factor essential for T helper-2 effector cell differentiation, endows mouse T-regs with the ability to suppress T helper-2 responses (38). Moreover, deletion of the transcription factor STAT3 (transcription factor required for T helper-17 differentiation) from mouse T-regs resulted in the development of fatal intestinal inflammation (39). Single cell RNA sequencing of skin T-regs revealed that mouse T-regs preferentially expressed high levels T helper-2 associated transcription factor aka GATA3 during homeostasis. GATA3 deletion from mouse T-regs resulted in exacerbation of T helper-2 cytokine secretion and fibrosis in the skin (40). Fibrosis is the manifestation of chronically activated fibroblasts that secrete excessive amounts of ECM and leads to an increase in the dermal layer. Moreover, in mouse models of atopic dermatitis, retinoid-related orphan receptor α (RORα) in skin T-regs is important for restraining allergic skin inflammation (41).

More recently, single cell RNA-sequencing on isolated T-regs from the skin and skin draining lymph nodes (SDLN) of mice revealed a transcriptionally distinct feature of skin T-regs (42). As compared to the SDLN T-regs, skin T-regs are transcriptionally enriched in pathways associated with TGFβ and integrin signalling. In addition, there was a significant increase in the expression of receptor-ligand pairs that link to T-reg-epithelial cell crosstalk. The distinct transcriptomic signature implies a preferential interaction of skin T-regs with the skin epithelium to exert tissue specific functions. Further stratifying T-reg populations into those derived from the dermis and the epidermis revealed strong transcriptomic homogeny between the two T-reg populations. However, there was a notable preferential expression of Itgb8 gene, which form the TGFβ activating integrin αvβ8, in the epidermal T-regs. Epidermal T-regs also showed increased SMAD2/3 phosphorylation in response to TGFβ stimulation as compared to those of the dermis and SDLN. The TGFβ – integrin αvβ8 axis plays a major role in tissue repair/remodelling in the context of injury and skin pathology (43). Interestingly, while skin T-reg was shown to facilitate epidermal barrier repair (44), activation of latent TGF-β in T-regs *via* the TGF-β/αvβ8 pathway was shown to delay epithelial repair efficiency. Instead, the TGF-β/αvβ8 pathway elicited innate immune protection against *Staphylococcus* aureus infection. This implies the functional heterogeneity of tissue T-regs and the potential effect of tissue cytokine milieu on non-lymphoid T-reg responses. The exact mechanism of how skin T-regs balance the kinetics of tissue repair and pathogen defense in anatomically distinct areas of the skin remain to be elucidated.

Though similar to colonic T-regs, skin T-regs have unique features which may imply tissue specific functions. For example, the mice skin exclusively consists of T-regs that are lacking Id3 (the inhibitors of DNA binding 3) when compared to lymphoid T-regs. The Id3 negative cutaneous T-regs express inhibitory markers and probably more suppressive (45). Another molecule, arginase 2, has been shown to be preferentially expressed in healthy human skin T-regs compared to the skin T-effs or circulating T-regs. Arginase 2 increases in human T-regs in metastatic melanoma, and reduces in T-regs from psoriatic skin. Decreased levels of arginase 2 renders T-regs dysfunctional to suppress T-eff cells and vice vera. Arginase 2 helps T-regs to maintain tissue T-reg signatures, to regulate inflammation and enhance metabolic fitness (46). ARG2 expression in Tregs was shown to attenuate mTOR activity with time after Treg activation.

In 2011, the Abbas lab unraveled how chronic or repeated exposure to self-antigen within tissues leads to an attenuation of pathological autoimmune responses. Using a model of cutaneous self-antigen expression, it was found that self-antigen specific T-regs migrate out of the thymus. Further T-regs activate, proliferate, and differentiate into more potent suppressors in the SDLN and then migrate to the skin to mitigate autoimmune and skin inflammation. A subset of T-regs also confer a memory phenotype and are maintained for long periods with enhanced suppression capacity (47). These results are consistent with reported differences between the skin resident T-regs and those that have migrated into the tissue. For example, half of the T-regs in an inflamed mice ear are migratory and have been shown to have higher levels of CTLA4 and Nrp1 and lower levels of CD25 and CD39 when compared to the resident T-regs (48). The diversity in gene expression and function points to the varying potential of T-regs in suppressing inflammation. Important questions arise from these observations: 1. Does the diversity of T-reg subsets suggest that they perform multiple non-overlapping functions in the skin? and 2. Are some of those cutaneous functions associated with the non-immune cells?

## Trafficking of cutaneous T-regs

As an example of its role as an immunological barrier, the human skin contains more T cells than the bloodstream. It is estimated that there are around 1 million T cells per square centimeter and 10 billion T cells in the entire skin (49). T-regs represent around 20% of the tissue resident CD4+ T cells in human skin and 20-60 percent in the mouse skin (4, 16, 41). Where do these T cells come from and how do they migrate to the skin?

The cutaneous migration of T-regs is important as the inability of T-regs to home to the skin is permissive of dermatitis and cutaneous inflammation (50). T-regs enter the skin is after post-natal day 6 (51), indicating that these cells are not required for the embryonic and early development this organ. However, there is a peak in the number of T-regs at post-natal day 13 when roughly 80 percent of CD4+ T cells are T-regs, which have migrated from the thymus (51). The significance of this peak is linked to the tolerance of commensal microbes (51). Following this peak there is a dip in the number of T-regs and a second peak at post-natal day 23. The importance of this second wave of T-regs is its association with the proliferation of hair follicle stem cells and subsequent growth of the hair follicle (16). Further, in steady state human skin resident memory T-regs reside in the epidermis and in the follicular epithelium in proximity to dermal dendritic cell (DDC) (52). It would thus be important to determine if there is an increase in a specific type of T-regs (nT-regs vs. iT-reg) given their differential representation in other organs in physiology and disease. Moreover, whether the cyclical changes observed in T-regs and T-cells in the skin is due to interconversion between natural and induced subtypes is yet to be addressed.

The migration of T-regs into the skin from the thymus and lymph nodes is guided by signals arising from the tissue resident cells and the corresponding expression of their cognate receptors on the T-regs. One class of migratory signals is the chemokine and chemokine receptor. The CCR4 chemokine receptor is robustly expressed by skin-homing T cells and the CCR4 ligand, TARC (Thymus and activation-regulated chemokine) is expressed by the endothelial cells of the cutaneous vasculature (53). Upon genetic ablation of CCR4 expression in T cells, homing of T cells to the skin is prevented (54). Cutaneous T-regs have also been shown to express CCR4, thus loss of CCR4 in T-regs, confers to them a competitive disadvantage with other CCR4 expressing T-cells to migrate into the skin and lungs. This leads to lymphocytic infiltration and severe inflammation in the skin (55).

Upon reaching the skin through the vasculature, cell adhesion molecules on the T-regs aid in binding the cell to the endothelium and promote extravasation. Surface expression of the ligands for E and P selectins found on endothelial cells is required for the exit of the T-regs from the circulation (3, 56). In a model of contact hypersensitivity, rolling of endogenous T-regs in dermal postcapillary venules was dependent on overlapping contributions of P- and E-selectin. However, after a repeated challenge, T-regs but not conventional CD4+ T cells became P-selectin independent. This was also supported by the reduced capacity of T-regs to bind P-selectin. Inhibition of E-selectin during a repeated challenge resulted in exacerbation of inflammation. This report suggested that there is a dynamic molecular shift in T-regs: the initial migration requires both P- and E-selectin whereas repeated challenge requires only E-selectin (57). The α-1,3-fucosyltransferase VII (FuT7) enzyme is responsible for catalyzing the formation of the carbohydrate determinants of E- and P-selectin. Consistent with this, the loss of FuT7 also reduced T-reg cell accumulation in the mouse skin, resulting in selective onset of severe cutaneous inflammation (50). Additionally, layilin (a C-type lectin-like receptor) is preferentially and highly expressed on a subset of activated Tregs in human skin (58). Layilin is induced by TCR-mediated activation (by IL-2 or TGF-β) and facilitates Treg adhesion in skin. Moreover, Layilin expression on T-regs limits their suppressive capacity (58).

Under homeostatic conditions, microbial-epithelial interactions also contribute to the migration of T-regs into the skin. The commensal bacteria of the skin microbiome play important roles in maintaining homeostasis (59). The tolerance of the skin commensal microbes is maintained by the cutaneous T-regs as they accumulate in the skin at postnatal days 6–13 (51). In the absence of this microbial interaction (such as the case of germ-free mice), a 20 percent reduction in the number of neonatal skin T-regs was reported (60). Furthermore, the microbiome induces the expression of CCL20 in the hair follicle cells and T-regs possess the receptor for CCL20 (i.e., CCR6) which aids in their retention near the hair follicle (60). CCR6 is a G protein-coupled receptor expressed on immature DCs, innate lymphoid cells, T-regs, Th17 cells and B cells (61). In human oral squamous cell carcinoma, CCR6 expressing T-reg were more suppressive with higher FOXP3 expression compared to CCR6 negative T-regs (62). Human skin T-regs express high levels of the memory markers such as CD27 and BCL-2, suggesting that they are effector memory T-regs (4). The expression memory markers can be a result of the interaction of T-regs with the skin microbiome.

## Unconventional roles of cutaneous T-regs: Barrier function and wound healing

The functional importance of cutaneous T-regs in supporting skin barrier integrity was shown in mouse models where T-regs were selectively impaired in their ability to migrate to the skin. These mutant mice spontaneously developed visible dermatitis accompanied by a dense dermal infiltration of leukocytes and thickening of the epidermis (50, 55). Even in the absence of immunogenic foreign antigens, the lack of constitutive cutaneous T-reg migration can results in progressive immune responses. This can result from the mounting of an immune response from self-antigens, or from the skin’s own microbiome or the environment, which normally do not elicit a strong inflammatory response.

It is increasingly recognized that the skin microbiome is an integral part of the skin barrier that influence many aspects of skin health (63) and shape the cutaneous innate and adaptive immunity (64). An important method by which the skin microbiome contributes to homeostatic immunity is *via* regulating the immigration of T-regs into the skin to establish tolerance (51). While early skin colonization of commensal is critical for establishing commensal-specific immune tolerance (51), skin T-regs are also involved in regulating commensal-specific T cell responses in relation to local immunity and tissue remodeling (65). Harrison et al. reported that Foxp3+ T-regs are localized in closed proximity to *S. epidermidis*-induced CD8+ T cells in murine epidermis. Reduced level of skin T-regs through conditional deletion of GATA-3 in Treg cells were shown to selectively unleash type-2 cytokines by commensal-specific, RORγt-committed type-17 cells. which may be licensed by alarmins associated with tissue damages (65). This data suggests a potential role of skin T-reg in regulating commensal-specific T cell plasticity, allowing the cells to exert pleiotropic immunity and tissue repair functions, while preventing tissue predisposition to inflammation.

In addition, recent insights have revealed the unconventional roles of immune cells, in not only protecting the wound site from infection but actively promoting restoration of tissue homeostasis. Upon breaching of the physical barrier of the skin the wound healing program is initiated and is comprised of three sequential and overlapping phases: inflammation, proliferation, and remodeling. The early phase of the wound repair process is the inflammatory phase, which is rapidly launched by damage-associated molecular patterns (DAMPs) and chemokines released by the cells in wound areas that recruit and activate innate immune cells and later adaptive immune cells. These immune cells are critical to fending off infections but they must be tightly regulated to avoid secondary damage to the tissue.

Though regulated by different chemokines produced from numerous cell sources in the skin, it is tempting to speculate that different T-reg sub-populations may also contribute to the sequential appearance and activity of the innate and adaptive immune cells during the wound healing process. This is based on the correlation with the sequential recruitment of T-regs in the wound healing program. For instance, CD25+ T-regs are increased at an early-stage post wounding (day 3) while ICOS+ and CTLA4+ T-regs are increased at later stages (Day 7) (66). Interestingly, early ablation of T-regs (2 day prior to wounding up to 5 days post wounding) leads to significantly slower wound closure rates. However, depletion of these cells at later stages (post wounding day 5 to 10) had no effect on wound closure kinetics (66). The early ablation of T-regs in the wounded skin leads to exacerbated production of IFNγ (66), which increases monocyte differentiation to M1 (pro-inflammatory) macrophages as well as their accumulation (67). A functional consequence of accumulating the M1 macrophage is a delay in the wound healing program (68). There are additional instances in which T-regs impact the wound healing program. For example, upon depletion of T-regs in the wounded mice, the suppression of the immune responses is absent which leads to impaired barrier restoration (44). T-reg cells were shown to preferentially suppress early accumulation of IL-17A CD4+ T cells at the site of injury. In these settings, the T-reg mediated control of IL-17-CXCL5-neutrophil axis of inflammation is absent which in turn prevents the differentiation of hair follicle stem cells into the epithelial keratinocytes to aid in reepithelialization (44). Notably, in their model, absence of Treg cells compromised epidermal barrier repair upon injury but did not alter skin barrier integrity under homeostatic condition.

**Figure 2:**
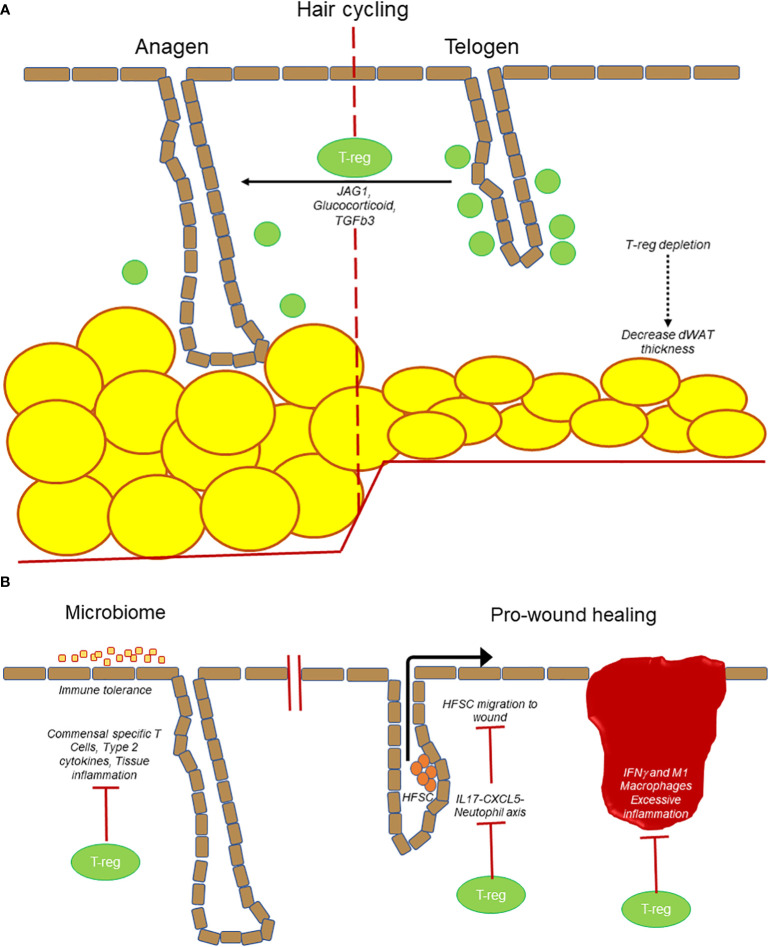
T-regs are at the center of many processes in the skin. **(A)** T-regs play a direct role in hair follicle cycling by promoting transition from telogen to anagen stage. Loss of T-regs also results in a decrease in dWAT thickness. **(B)** T-regs establish immune tolerance and prevent microbiome specific inflammation. T-regs also promote wound healing by suppressing IL17-CXCL5-neutophil axis to promote hair follicle stem cell (HFSC) migration. T-regs prevent excessive inflammation (IFNγ and M1 macrophages) to prevent delayed wound healing.

Differentiation of dermal fibroblast into myofibroblasts (ECM producing activated fibroblast) is another key event in tissue regeneration which requires tight regulation. In this context, T-regs prevent Th2 responses in the skin to suppress myofibroblast activation and thus chronic reduction of T-regs leads to increased TH2 cytokine production in skin, leading to persistent fibroblast activation and fibrosis (40). In a recent study in 2021, the number of IL-10^+^ and TGF-β^+^ T-regs were shown to increase after the topical application of 4% sodium dodecyl sulfate mediated skin barrier disruption in the mice (69). IL-10^+^ and TGF-β^+^ T-regs might play roles in suppressing inflammation and/or facilitate the barrier repair. Altogether, these observations highlight the importance of cutaneous T-regs as an integral component in maintaining skin immune homeostasis, thereby sustaining a healthy skin barrier function and support/regulating tissue regeneration (**Figure 2**)

The reparative roles of T-regs are not only limited to the skin as it has also been reported to promote healing in the lungs and muscle (70, 71). A distinct population of T-regs can help in muscle repair by promoting satellite cell function and muscle repair by amphiregulin. A similar mechanism was reported in lung tissue repair by amphiregulin belonging to the same family of EGFR (70). Although there are very few T-regs in the brain, recent work (72) suggests that these cells play a role in restoring neurological homeostasis after ischemic stroke. These anecdotes suggest that we have just scratched the surface of understanding the full extent the role of T-regs in tissue repair throughout the body.

## Hair follicle cycling

Hair follicles are “mini-organs” that undergo continuous cycles of regeneration. Interestingly, an emerging area of interest is the regulation of hair follicles by immune cells. At telogen stage, T-regs localize near the hair follicle stem cell niche known as the bulge region. In the well-established model of depilation-induced hair follicle regeneration, it was found that T-regs preferentially express the Notch ligand, Jagged1, which activates the Notch target genes transcripts on the hair follicle stem cells to initiate the transition from telogen to anagen (growth) stage. Subcutaneous administration of microbeads coated with Jag1-Fc was able to partially rescue HFSC activation and induction of anagen in the absence of Tregs. The result of Notch and Jagged1 interaction results in the proliferation and differentiation of hair follicle stem cells (16). More recently, it was reported that glucocorticoid signaling in T-regs is important in stimulating TGFβ3 production that can signal to the nearby hair follicle stem cells to enter anagen (73). Altogether these findings suggest that T-regs have direct unconventional functions in the skin to regulate the regeneration of the hair follicles (**Figure 2**).

## Impact of T-regs *via* its regulation of other immune cells with unconventional functions in the skin

Other components of the skin undergo cyclic regeneration such as the dermis, adipose tissue, and epidermis, and it is tempting to propose that the cyclical nature of T-regs likewise regulates these processes either directly or through their modulation of other immune cell activities. It is well established that T-regs suppress various immune cells from generating an autoimmune response or ameliorating the inflammatory activity after infection or injury or antibody response. Thus, T-regs can function at a secondary level to control adaptive immune cells that in turn have novel functions in the skin (**Figure 2**).

### Non-classical functions of adaptive immune cells

CD4+ and CD8+ T-cells are present in the skin at lower frequencies than macrophages, but during wound healing, infection, or autoimmunity, the numbers of T cells skyrocket. T cells have been found to affect numerous cells of the skin including dermal fibroblasts, keratinocytes, and hair follicles during pathological conditions (74, 75). The interaction of T cells with different compartments and cell types in the skin can be through the secretion of various cytokines. For example, some cytokines mediate interactions between the T-cells and fibroblasts- thereby affecting the activation status of the former:

- IL22 is a cytokine produced by subsets of T cells and has been linked to fibroblast function during wound healing. Both *in vivo* and *in vitro* evidence has revealed that IL22 in wounds displays severe defects in the dermal compartment and inhibits the ability of fibroblasts to produce ECM and differentiate into myofibroblasts (76).
- Similarly, in the GVHD-SSc mice that exhibit fibrotic skin, it was found that ICOS+ TFH-like (Inducible T-cell co-stimulator+ T Follicular Helper-like) cells produce IL21 and MMP 12, which cause activation of fibroblasts that lead to the pathology. Upon administration of anti-ICOS antibody or IL21 neutralization in GVHD-SSc, skin fibrosis was reduced (77).
- IL13 secreted by CD8 T cells has been shown to increase the ECM production from dermal fibroblasts (78). Similarly, when conditioned media from CD8^+^ CD28^-^ T cells isolated from patients with the skin fibrotic disease scleroderma was added to dermal fibroblasts, there was an increase in collagen and fibronectin production. This effect was inhibited by the pre-treatment of IL13 antibody suggesting IL13 production from CD8 T cells causes activation of fibroblasts (79).
- TGFβ is a well-known cytokine that causes fibroblast activation and can be produced by various cell types in the skin. Conditioned media collected from the CD4 T cells from the skin of burn wound patients had significantly higher TGFβ levels compared to the normal subjects. Upon treatment of this conditioned media on fibroblasts, an increase in cell proliferation, collagen synthesis, smooth muscle actin levels, and collagen contraction was observed (80).

Interestingly, depletion of T-regs leads to an increase in Th2 cytokine production in the skin and the spontaneous activation of fibroblasts, which results in dermal fibrosis (40). T-reg depletion in the bleomycin-induced fibrosis model likewise exacerbated the fibrotic phenotype. Specific deletion of the transcription factor GATA3 in T-regs phenocopied the depletion of T-regs in terms of Th2 cytokine production and enhanced fibrosis. Moreover, antibody neutralization of IL4, the primary cytokine associated with Th2 type inflammation, augmented the fibrotic phenotype.

A major question in fibrogenesis is the source of these activated fibroblasts. Using a lineage tracing technique in bleomycin-treated mice, John Varga’s group demonstrated that the dermal adipocytes undergo an adipose to mesenchymal transition (AMT) (81). The resulting adipose-derived fibroblasts were actively producing collagens and contributing to fibrogenesis. This is consistent with observations in the fibrotic skin of both mouse and patients with scleroderma where an increase in the dermal layer is accompanied by a decrease in the dWAT layer (81–86). Interestingly, the study mentioned above by Rosenblum’s group (40), also noticed a decrease in the dWAT layer upon depletion of T-regs and was more pronounced upon bleomycin treatment. These reports imply a possible role of T-regs in the regulation of the dWAT layer. The histologic images of the skin section of the scurfy mice, which has complete loss of immunosuppressive activity of T-regs – display loss of dWAT, a phenotype that is often overlooked (87–91). In addition, the transient loss of T-regs also results in a decrease in the dWAT layer and an increase in the thickness of the fascia layer (92). In summary these data suggest that the T-regs in the skin prevent spontaneous inflammation, development of fibrosis, loss of dWAT layer and thickness of fascia layer. Nevertheless, questions remain about the role(s) that T-regs or Th2 inflammation play in the regulation of dWAT, whether these signals mediate an AMT *in vivo*, and if there more direct roles that T-regs can play, as reported in the case of hair follicle stem cells (16).

In addition to T-cell-derived cytokines, B cells have been shown to activate fibroblast in a contact-dependent manner. Co-culturing experiments revealed that B cells are capable of stimulating collagen secretion by dermal fibroblasts which is enhanced by B-cell activating factor (BAFF) (93). Likewise, a co-culture of circulating B cells and dermal fibroblasts isolated from SSc patients induced IL-6, TGF-β1, CCL2, and collagen secretion, as well as alpha-SMA, TIMP1, and MMP9 expression in dermal fibroblasts (93). Similarly, upon antibody mediated depletion of B cells in the Tight Skin model of systemic sclerosis, there was a significant reduction of fibrosis (94). Altogether, these results suggest that CD4 T+, and CD8 T+ can activate fibroblast *via* secreted factors and B cells achieve a similar response in a contact-dependent manner.

The thickening of the epidermal layer (epidermal hyperplasia) because of increased proliferation of the keratinocytes is a hallmark of the wound healing program as well as one of the major signs of diseased skin during pathological inflammation. The coincident observation of epidermal hyperplasia and inflammation suggest causal relationship. In fact, *in vitro* studies on epithelial keratinocytes suggests that IL22, which is often sourced from T-cells, can enhance the proliferative and migratory capacity of epidermal keratinocytes while their differentiation is repressed (95, 96). Further, it was reported that a subpopulation of lesional psoriatic T lymphocytes can enhance the proliferation of keratinocytes *in vitro* (97). Apart from the proliferation of keratinocytes apoptosis is associated with the pathogenesis of eczematous disorders. T cell infiltration leads to keratinocyte apoptosis by Fas ligand and Fas receptor interaction and increases susceptibility to INF-g mediated apoptosis (98). Another mode of inducing keratinocyte apoptosis is through the secretion of granzyme B from CD8 T cells (99). These results suggest that T cells can cause both proliferation and apoptosis of keratinocytes in different settings.

These findings suggest that T-regs have non-classical roles owing to their regulation of T and B-cells that are themselves increasingly recognized for unconventional activities (**Figure 2**). Given the heterogeneity of each of these cell types, it is likely that they will exhibit diverse activities, and many more novel cutaneous functions will be uncovered in both physiological and pathological scenarios.

### Non-classical functions of Innate immune cells

In addition to the adaptive immune cells, the innate immune cells likewise impact homeostasis of various compartments of the skin. These innate immune cells are also regulated by T-regs and form another indirect link between T-regs and skin homeostasis (**Figure 2**). Langerhans cells (LCs) perform barrier functions by forming tight junctions with keratinocytes in the epidermis and scout the surroundings for potential pathogens (100). They are also involved in scavenging apoptotic keratinocytes, a process necessary for the maintenance of immune tolerance in the epidermis (101) Under stress, specific regions of the HF secrete different cytokines that differentially influence LC activity. The S1P1 + bulge cells secrete CCL8 to prevent the influx of LC during stress thereby protecting the stem cell niche. The isthmus and infundibular regions of the hair follicle recruit LCs by the production of CCL2 and CCL20. In fact, it was shown that in the absence of HF stem cells, Langerhans cells do not repopulate the epidermis after LC depletion signifying the cross-talk between HFs and the LC population (102). Studies on human skin have shown that skin resident memory T-regs residing in the epidermis and in the follicular epithelium are in close proximity to CD1a+ LC and CD1c/BDCA 1+ dermal dendritic cell (DDC) population in steady-state (52). As T-regs are also in close proximity to the hair follicle stem cell region, it would be interesting to ascertain whether they also influence LC indirectly *via* hair follicles or directly by cell-cell contact.

T-regs regulate macrophages mainly through IL10, which is crucial for immune tolerance (103, 104). T-regs are also capable of inducing macrophage apoptosis by the Fas/Fas ligand pathway during septic shock (105). Therefore, its effect on macrophages endows T-regs with the ability to influence hair follicle cycling since macrophages have been shown to be responsible for the induction of hair follicle anagen phase and regression phase by producing different cell signaling molecules such as Wnt and FGF (9, 106). As mentioned previously, apoptosis of macrophages releases Wnt7b and 10a and induces anagen phase of the hair cycle. Consistent with this, inhibition of macrophage apoptosis or Wnt signaling, results in a delay in the entry of the hair follicle into anagen (8). Additionally, oncostatin M (OSMR), released by a subset of TREM2+ macrophages during the telogen phase, contributes to the maintenance of hair follicle stem cell quiescence during the telogen phase (107). Consequently, ablation of OSMR beta or STAT5 can lead to early anagen induction. Interestingly, there is an inverse correlation of T-regs and macrophages during the hair cycle suggesting that T-regs indirectly regulates this follicular regenerative process through its control of macrophages. However, the direct link between T-regs and macrophages in the skin and their effect on hair follicle cycling remains to be established.

Another interaction that has thus far been reported only in inflammatory conditions is the crosstalk between T-regs and mast cells. In these scenarios T-regs have a dual effect on mast cells. T-regs can inhibit mast cell degranulation and also stimulate the production of IL-6, a cytokine with pleiotropic effects ranging from mediating local and systemic inflammation, tumorigenesis and autoimmune diseases (108). Role of Mast cells (MC) in hair follicle homeostasis came to light from a study in lesional alopecia areata (109). It was reported that MCs in alopecia areata switch from immune-inhibitory to pro-inflammatory phenotype suggested by the decreased TGFβ1, IL-10 and increased Tryptase immunoreactivity. Further, upon cyclosporine stimulated and depilation induced anagen, MC degranulation was observed during the early stages of anagen. The secretome of mast cells which contain numerous cytokines such as ACTH (adrenocorticotrophic hormone), substance 48/80, and the neuropeptide substance P were all able to induce anagen in mice skin (110). Similarly, induction of anagen was significantly delayed by MC degranulation inhibitors. However, the direct connection between T-regs and mast cells in the skin and their effect on hair follicle cycling remains to be established. In a mouse skin transplantation model where anti-CD154 and donor-specific transfusion (DST) was used to induce tolerance, a marked link between MCs and T-regs was observed through IL-9. IL 9 is a mast cell growth factor that can enhance the survival of primary MC, and induce the production of inflammatory cytokines, mast cell proteases, and high-affinity IgE receptors (FceR1 alpha). Upon activation, T-regs up-regulated IL9 production and a higher level of IL 9 was observed in tolerant grafts as opposed to rejected grafts. This tolerance could be due to the suppression of alloreactive CD8 T cells *via* the T-reg-IL9-MC axis (111, 112).

## Future prospective

**Figure 3:**
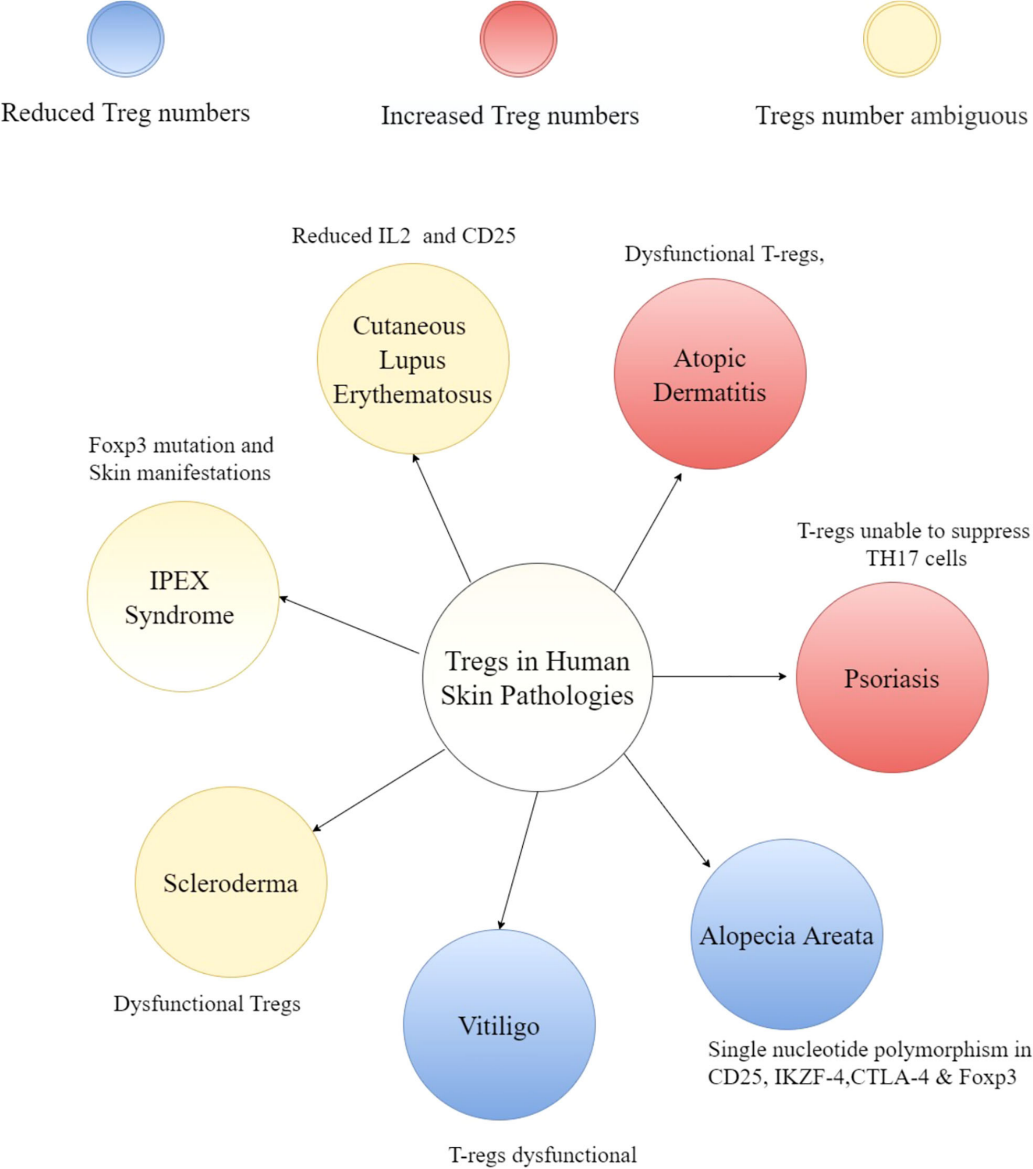
Overview of T-regs in human skin pathologies. A hub and spoke diagram summarizing the potential impact of T-regs and cytokines influencing the niche disease state.

As discussed above, perturbations in T-regs or its downstream effectors often results in skin phenotypes in mice. This correlates with many human skin diseases (**Figure 3**) that are accompanied by changes in T-reg numbers or activity such as scleroderma (113–116), alopecia (117–119), psoriasis (120–123), atopic dermatitis (27, 124, 125) and vitiligo (126–128). Likewise, diabetic patients have compromised T-regs (129–131) which likely contributes to their chronic non-healing wounds. The status of T-regs in inflammatory conditions and their utility in combating scenarios such chronic wounds is largely unknown and an area that is ripe for investigation. As the major node of regulation of the immune responses in various tissues including skin, it is not too surprising that many cutaneous diseases are marked by perturbations in T-regs or its downstream effectors, thereby suggesting a causal role in pathogenesis. The role of T-regs in human diseases has been extensively covered elsewhere (132, 133), so we will only give a brief overview of this topic.

Though there is a debate about whether the number of T-regs are altered in the fibrotic skin disease scleroderma, it is clear that these cells are dysfunctional (134, 135). In the case of alopecia areata, in which the hair follicles lose their immune privilege and are targeted by CD8+ T-cells, the number of T-regs are reduced (118) and unable to perform suppressive functions *in-vitro* (136). Genome-wide association studies (GWAS) in alopecia revealed single-nucleotide polymorphisms (SNPs) in regions encoding Treg signature genes such as CD25, the ikaros family member Eos (IKZF4), cytotoxic T-lymphocyte antigen 4 (CTLA-4) and Foxp3 (117, 119). These SNPs can possibly render T-regs dysfunctional. Dysfunctional T-regs have also been reported in psoriatic plaques which are unable to suppress TH17 responses (122). Further studies have revealed that T-regs in psoriasis can differentiate into IL-17 producing cells (137, 138). Similarly, T-regs can produce TH2 cytokines and contribute to the progression of atopic dermatitis (139). Tregs have also been shown to attenuate skin inflammation in several mouse models of atopic dermatitis (41, 140, 141). These results strongly suggest role of T-regs in various skin pathologies and likelihood of therapeutic window for many inflammatory skin diseases. For example, IL-2 treatment to boost T-regs have shown promising results of hair regeneration in patients with alopecia areata (142). Similarly, IL-2 mediated augmentation of T-regs reduces skin fibrosis in some patients suffering from graft-versus-host disease (GVHD) (143, 144). Interestingly, autologous hematopoietic stem cell transplantation which led to increase in the number of T-regs (along with higher expression of CTLA-4 and GITR on Tregs) resulted in clinical improvement in systemic sclerosis patients (145).

In the last two decades the unconventional roles of T-regs in skin and other tissues have been unraveled in both physiology and disease. Given the phenotypic diversity of T-regs and the ability to control both the innate and adaptive arms of immunity, we expect in the next few years a plethora of new and exciting functions of T-regs would be uncovered. The increased understanding of the diverse activities of T-regs will also open novel avenues for therapeutic intervention of many common inflammatory skin diseases. Lastly, development of T-reg based immunotherapies could potentially improve the quality of life in patients suffering from skin inflammatory pathologies.

## Author contributions

EH, PP, C-CC, HJ, AM, and CJ wrote different sections of the review article. Order of authorship was determined based on the amount written. EH and CJ organized the review, assigned authors for each section, and revised the manuscript. All authors contributed to the article and approved the submitted version.

## Funding

Research in the Jamora Laboratory is supported by core funds from the Institute for Stem Cell Science and Regenerative Medicine (inStem), Bellary Road, Bangalore, India and grants from the Department of Biotechnology of the Government of India (BT/PR8738/AGR/36/770/2013) and (BT/PR32539/BRB/10/1814/2019). EH was supported by an Indian Council for Medical Research (ICMR) Senior Research Fellowship. Animal studies in the Jamora lab is partially supported by the National Mouse Research Resource (NaMoR) grant BT/PR5981/MED/31/181/2012;2013-2016;2018 and 102/IFD/SAN/5003/2017-2018 from the Department of Biotechnology.

## Conflict of interest

Authors CC and AM were employed by company Unilever.

The remaining authors declare that the research was conducted in the absence of any commercial or financial relationships that could be construed as a potential conflict of interest.

## Publisher’s note

All claims expressed in this article are solely those of the authors and do not necessarily represent those of their affiliated organizations, or those of the publisher, the editors and the reviewers. Any product that may be evaluated in this article, or claim that may be made by its manufacturer, is not guaranteed or endorsed by the publisher.
